# Prolonged onset and duration of action of rocuronium after accidental subcutaneous injection in a patient with chronic renal failure—a case report

**DOI:** 10.1186/s40981-021-00421-3

**Published:** 2021-02-27

**Authors:** Akira Doshu-Kajiura, Junko Suzuki, Takahiro Suzuki

**Affiliations:** Department of Anesthesiology, Nihon University, School of Medicine, 30-1, Oyaguchi, Kami-cho, Itabashi-ku, Tokyo, 173-8610 Japan

**Keywords:** Acceleromyograph, Hemodialysis, Pharmacodynamics, Rapid sequence induction, Rocuronium, Subcutaneous injection, Sugammadex

## Abstract

**Background:**

Drugs administered subcutaneously have delayed onset and prolonged duration of action versus those given intravenously. Since the duration of action of rocuronium is prolonged in patients with renal dysfunction, subcutaneous administration of rocuronium to such patients might significantly prolong its effect.

**Case presentation:**

A 51-year-old female with chronic renal failure was accidentally administered 1.04 mg/kg rocuronium subcutaneously. Marked prolongation of onset and duration of action of rocuronium were detected on acceleromyography. Slow development of the neuromuscular block was still observed at 100 min after injection. Administration of 4.5 mg/kg sugammadex at 140 min after rocuronium injection facilitated recovery from a train-of-four (TOF) count of 2 to a TOF ratio of 100% within 5 min. No symptoms of postoperative recurarization and upper airway obstruction were observed.

**Conclusion:**

Neuromuscular monitoring is necessary to evaluate the progress and depth of neuromuscular block, particularly when rocuronium is inadvertently administered subcutaneously.

## Background

Accidental subcutaneous administration of muscle relaxants results in delayed onset and prolonged effect [1, 2]. Although the pharmacodynamic profiles of subcutaneously administered rocuronium are unclear, its duration of action would be significantly prolonged in patients with renal failure because it is partially excreted via the kidneys [3]. We report a case with renal failure in whom neuromuscular blockade was prolonged after subcutaneous administration of rocuronium. Neuromuscular blockade was quantitatively monitored by acceleromyograph and was successfully reversed with sugammadex.

## Case report

**Fig. 1:**
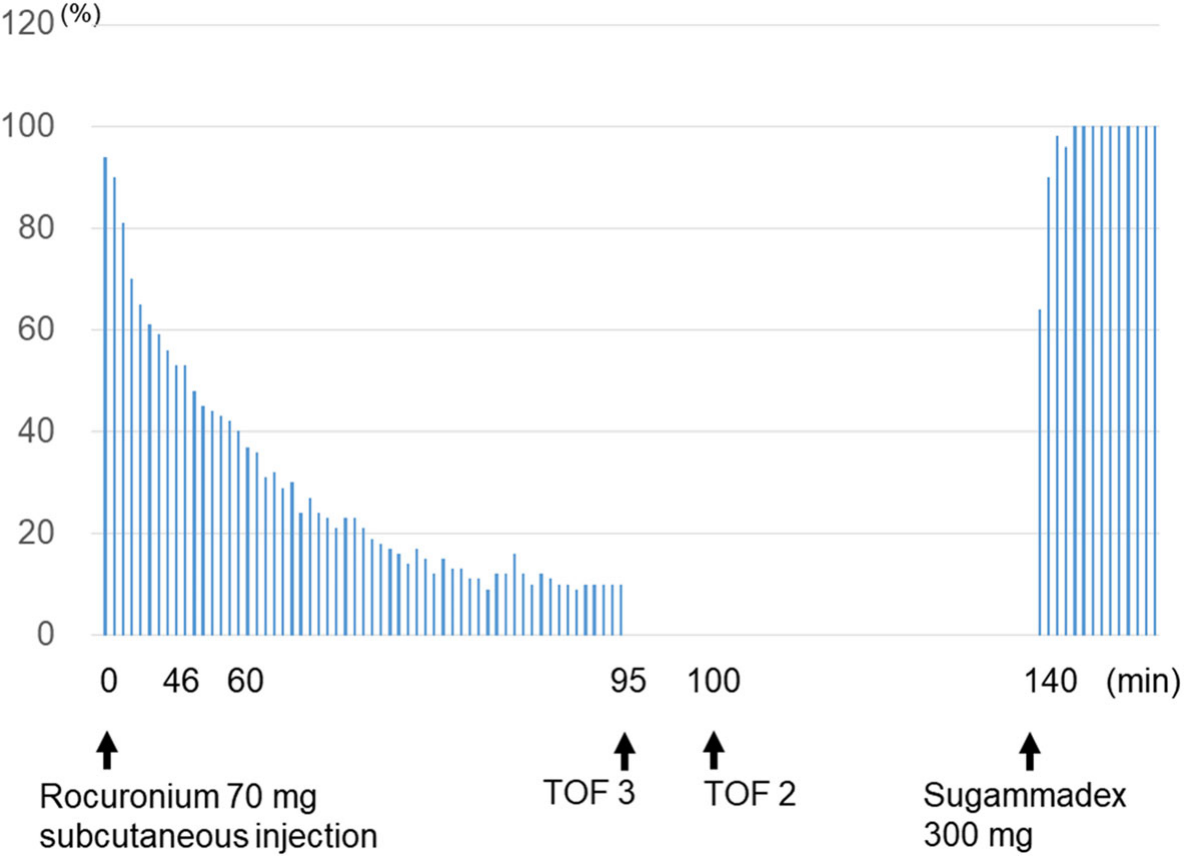
Train-of-four ratio after subcutaneous administration of rocuronium. The vertical bars show progression of the TOF ratio. Slow development of the neuromuscular block was seen after subcutaneous injection of rocuronium. The fourth twitch of a train-of-four stimulation was not detected after 95 min. Train-of-four count was 3, 95 min after administration of rocuronium, and it remained 2 until the end of the surgery. Sugammadex rapidly reversed the neuromuscular block to a TOF ratio of 100%

Written informed consent for publication of this report was obtained from the patient. A 51-year-old woman (164 cm, 67 kg) with a diagnosis of acute cellulitis of the left knee was scheduled for emergency debridement under general anesthesia. The patient has been on hemodialysis for 4 years due to diabetic nephropathy. A 20 G intravenous (IV) catheter was placed in the right median cubital vein in the emergency room and infusion of lactated Ringer’s solution was commenced using an infusion pump. When she arrived at the operation room, the speed of infusion was as expected and backflow of blood was observed on interrupting the infusion. After pre-oxygenation with 100% oxygen via a face mask and a continuous infusion of remifentanil 0.3 μg/kg/min for 5 min, the patient received IV propofol 100 mg and rocuronium 70 mg (1.04 mg/kg) for rapid sequence induction. However, the patient remained completely conscious 90 s later, when the anesthesiologists detected significant swelling around the site of insertion of the peripheral IV catheter, without any complaint of pain at the site. Her contralateral arm, which had a hemodialysis shunt, and her lower limbs, which had marked edema, were unsuitable for insertion of a new peripheral intravenous line. Therefore, anesthesia was induced with inhalation of 8% sevoflurane via a face mask. After loss of consciousness, the patient’s trachea was intubated under spontaneous ventilation following nebulization with 8% lidocaine. Thereafter, a central venous catheter was inserted in her right internal jugular vein. Anesthesia was maintained with 0.9–1.2% end-tidal sevoflurane and a continuous infusion of 0.1–0.3 μg/kg/min remifentanil via the central venous catheter. The patient received no more rocuronium during anesthesia. Ventilation was adjusted to maintain end-tidal carbon dioxide tension within the range of 35–40 mmHg. Body temperature was kept at > 36.0 °C using a warming blanket. Subsequently, neuromuscular monitoring at the left adductor pollicis muscle was initiated using an acceleromyograph (TOF Watch™, Nihon Kohden, Tokyo, Japan). The ulnar nerve at the wrist was stimulated using square-wave stimuli of 0.2-ms duration, which was delivered in a train-of-four (TOF) mode at 2 Hz every 15 s. All data were recorded in the electronic anesthetic chart (CAP-2000, Nihon Kohden) on a minute-by-minute basis. The TOF ratio observed 20 min after injection of rocuronium was 94%, gradually decreasing to 50% after 46 min and 25% after 60 min. Thereafter, it could not be displayed because of progression of neuromuscular block, and instead, the TOF counts were indicated on the monitor. TOF counts of 3 and 2 were observed at 95 min and 100 min after rocuronium administration, indicating that rocuronium-induced neuromuscular block was still progressing (Fig. 1). A TOF count of 2 was repeatedly observed until the surgery was completed 140 min after rocuronium injection. Sugammadex 300 mg (4.5 mg/kg) facilitated the recovery of the TOF ratio to 90% within 2 min and to 100% within 5 min. Once a TOF ratio of 100% was found to be ongoing for 10 min, anesthesia was discontinued. The patient immediately and fully regained consciousness and respiration and was uneventfully extubated. We subsequently monitored the patient in the intensive care unit for 6 h, with no symptoms of upper airway obstruction or desaturation being observed.

## Discussion

The onset of action of intravenous rocuronium 1 mg/kg, i.e., the time required to reach the maximal depression of T1 at the adductor pollicis muscle, is 77 s [4]. Rapid onset is a specific characteristic of rocuronium compared to other non-depolarizing neuromuscular blocking agents. However, in our patient, the neuromuscular block developed slowly over 100 min after accidental subcutaneous injection of 1.04 mg/kg rocuronium. The pharmacokinetics of intravenously administered rocuronium can be explained by the three-compartment model [5]. However, because the clearance of subcutaneously administered rocuronium is unknown, it is difficult to predict the real onset and duration of action of rocuronium administered subcutaneously. Molecules of subcutaneously administered drugs might be slowly absorbed into the systemic circulation, taking a longer time to reach the effect sites, resulting in delayed onset and prolonged duration of action as compared to intravenous administration. Since the onset of action depends on the diffusion speed of the drug from extravascular sites, the patient’s health comorbidities might considerably influence the absorption. Patients with peripheral circulatory disturbances and arteriosclerosis might have slow absorption of rocuronium from subcutaneous regions into the blood, and hence, might unexpectedly exhibit slower onset and longer progression of the action of neuromuscular block. Navare and colleagues reported a similar case that had end-stage renal disease and was given an incidental subcutaneous injection of 1.16 mg/kg rocuronium [2]. Unfortunately, although they only observed neuromuscular block qualitatively and assessed TOF counts of the thumb to ulnar nerve TOF stimulation using a peripheral nerve stimulator, it took 39 min for the initial decline in TOF count from 4 to 3 in their patient. As can be expected, peripheral circulatory failure due to diabetes mellitus and arteriosclerosis might significantly slow the absorption of rocuronium from the subcutaneous space. Rocuronium is mainly excreted in bile via the liver [6] and is partially eliminated in urine via the kidneys [3]. Therefore, the clearance of rocuronium decreases by about 20% in patients with chronic renal failure. The slowed elimination of rocuronium from the body might have also been involved in delayed recovery from the rocuronium-induced block in this patient.

Since we quantitatively monitored rocuronium-induced neuromuscular block using acceleromyography, we were able to recognize depressed neuromuscular function, as seen by the TOF count of 2 at the completion of surgery, and could adequately reverse the moderate depth of neuromuscular block with 2 mg/kg of sugammadex. Reportedly, sugammadex 2 mg/kg is sufficient to antagonize rocuronium-induced moderate neuromuscular block in hemodialysis patients [7]. However, it should be remembered that neuromuscular monitoring only indicates the action of rocuronium molecules that have diffused into the neuromuscular junction, and does not demonstrate the amount of rocuronium remaining in a subcutaneous depot. In our case, we administered 4.5 mg/kg of sugammadex, nearly equal to the recommended dose for recovery of a deep neuromuscular blockade, although the TOF count was 2 at the end of surgery. TOF ratio and TOF count continued to decline at that time, so we could not rule out the possibility to change TOF count from 2 to 1 or more deeper level. It would, therefore, be better to administer a larger than usual dose of sugammadex to patients with inadvertent subcutaneous rocuronium injection in order to prevent residual neuromuscular block and recurarization.

In conclusion, because the action of rocuronium injected subcutaneously might vary between patients, making it extremely difficult to predict its onset and duration of action, rigorous quantitative neuromuscular monitoring, adequate dosing with sugammadex, and strict monitoring of respiratory function in the postoperative period can contribute to safe management of patients with inadvertent subcutaneous injection of rocuronium.

## Data Availability

Not applicable.
